# Targeting ERRs to counteract age-related muscle atrophy associated with physical inactivity: a pilot study

**DOI:** 10.3389/fphys.2025.1616693

**Published:** 2025-07-07

**Authors:** Roberto Bonanni, Angela Falvino, Antonio Matticari, Anna Maria Rinaldi, Giovanna D’Arcangelo, Pierangelo Cifelli, Riccardo Iundusi, Elena Gasbarra, Virginia Tancredi, Ida Cariati, Umberto Tarantino

**Affiliations:** ^1^ Department of Biotechnological and Applied Clinical Sciences, University of L’Aquila, L’Aquila, Italy; ^2^ Department of Biomedicine and Prevention, “Tor Vergata” University of Rome, Rome, Italy; ^3^ Department of Orthopaedics and Traumatology, “Policlinico Tor Vergata” Foundation, Rome, Italy; ^4^ Department of Systems Medicine, “Tor Vergata” University of Rome, Rome, Italy; ^5^ Centre of Space Bio-Medicine, “Tor Vergata” University of Rome, Rome, Italy; ^6^ Catholic University “Our Lady of Good Counsel”, Tirana, Albania

**Keywords:** ERRs, skeletal muscle, aging, physical inactivity, physiology, biomarkers

## Abstract

**Introduction:**

Estrogen-related receptors has been suggested as a potential therapeutic target to counteract muscle decline associated with aging or inactivity, being known to regulate mitochondrial function and cellular respiration by up-regulating key factors in muscle responses to exercise. This study aimed to evaluate the targeting of ERRs in myoblasts isolated from the skeletal muscle of inactive women by assessing the metabolic and expression changes associated with its activation.

**Methods:**

Twenty women undergoing hip arthroplasty for coxarthrosis were enrolled and divided into an active group (n = 10) and an inactive group (n = 10) based on self-reported physical activity. During surgery, muscle biopsies were taken for histological and western blotting analysis, measuring the expression levels of NADPH oxidase 4 (NOX4), sirtuin 1 (SIRT1), peroxisome proliferator-activated receptor gamma coactivator 1-alpha (PGC-1α), estrogen related receptor alpha (ERRα), and fibronectin type III domain-containing protein 5 (FNDC5). Primary cultures of myoblasts were set up from the muscle tissue of inactive women and treated with the ERRs agonist, SLU-PP-332, for subsequent qualitative and quantitative investigations. In addition, myoblasts were differentiated into myotubes for 15 days, and the success of differentiation was evaluated by immunofluorescence analysis.

**Results:**

Clinical and instrumental evaluation showed less functional limitation, higher handgrip strength values, and significantly reduced visual analogue scale scores in active subjects, in association with a significant increase in muscle fiber diameter. In addition, significantly higher expression of NOX4, concomitant with reduced levels of SIRT1, PGC-1α, ERRα, and FNDC5, was detected in the muscle tissue of inactive women. Interestingly, SLU-PP-332 treatment promoted down-regulation of NOX4 and upregulation of SIRT1, PGC-1α, ERRα, FNDC5, Akt, and B-cell lymphoma 2 (Bcl-2) in myoblasts, reducing cytotoxicity, oxidative stress, and senescence, as well as increasing levels of reduced glutathione. Furthermore, SLU-PP-332 treatment promoted abundant myotube formation, positively influencing cell differentiation.

**Discussion:**

Targeting ERRs could represent a promising therapeutic strategy to counteract muscle atrophy in elderly and sedentary subjects. However, further studies are needed to clarify the molecular mechanisms involved and explore the impact of ERRs activation on muscle metabolism.

## 1 Introduction

Aging involves dramatic structural and metabolic changes in the musculoskeletal system, leading to muscle atrophy and compromised muscle strength and function (Zhang et al., 2025b). Muscle cells are known to play an important role in muscle decline during aging, as their content has been suggested to be a strong predictor of muscle fibre size in elderly subjects (Brack et al., 2007; Huo et al., 2022). In fact, Verdijk et al., analyzing muscle biopsies from elderly subjects, found a significant reduction in the number of satellite cells associated with a reduction in type II fibre size (Verdijk et al., 2014). This condition of muscle decline is often associated with musculoskeletal pain, which promotes a sedentary lifestyle, further exacerbating muscle tissue depletion (Tarantino et al., 2022). Indeed, reduced physical activity levels are a significant risk factor for disability during aging, as they are linked to an increased risk of falls and mortality (Mo et al., 2023). On the other hand, exercise is known to promote numerous physiological adaptations in muscle tissue that collectively preserve its structure and function (Falvino et al., 2024). Particularly, exercise has been identified as the best strategy to prevent frailty, improve sarcopenic status and physical function in the elderly, as well as to increase muscle strength, aerobic capacity, and endurance (Landi et al., 2014; Cariati et al., 2021). Furthermore, resistance exercise has been proposed as a countermeasure to increase the number of satellite cells and reverse muscle atrophy during aging (Verdijk et al., 2014). However, the molecular mechanisms underlying these adaptations are extremely complex and still partially unexplored, highlighting the need for substantial research to identify potential therapeutic targets that could counteract musculoskeletal deterioration in the elderly.

In this context, fibronectin type III domain-containing protein 5 (FNDC5) has been identified as a precursor of the hormone irisin, whose release significantly increases in response to exercise, influencing the metabolism of several organs and systems (Maak et al., 2021; Waseem et al., 2022). In addition, the overexpression of sirtuin 1 (SIRT1), a NAD^+^-dependent deacetylase, which occurs in response to exercise, appears to promote the increase in the number of myonuclei in muscle fibers, stimulating muscle repair processes and inducing muscle hypertrophy (Radak et al., 2020). Undoubtedly, the upregulation of SIRT1 represents a key step in the physiological adaptations to exercise, as it is involved in regulating mitochondrial function (Menzies and Hood, 2012) and reducing the production of reactive oxygen species (ROS) by NADPH oxidase 4 (NOX4), a major inducer of oxidative stress (Dasgupta et al., 2020). In fact, mitochondrial dysfunction associated with the downregulation of SIRT1 seems to represent a key event in muscle atrophy during aging, highlighting the important role of this critical regulator of muscle metabolism (Yang et al., 2024).

Notably, SIRT1 is known to promote the activation of peroxisome proliferator-activated receptor gamma coactivator 1-alpha (PGC-1α), a member of a family of transcriptional coactivators that plays a central role in regulating cellular energy metabolism by stimulating mitochondrial biogenesis (Liang and Ward, 2006). The importance of PGC-1α in skeletal muscle metabolism has been highlighted by Cannavino et al., who observed the preservation of muscle mass in mice subjected to limb unloading that overexpressed PGC-1α, suggesting a therapeutic potential for compounds capable of stimulating its expression (Cannavino et al., 2014). Interestingly, PGC-1α forms a transcriptional complex with the estrogen-related receptor alpha (ERRα), characterized by constitutive ligand-independent activity induced by exercise, promoting the activation of exercise-responsive genes (Giguère, 2008). This transcriptional axis, composed of PGC-1α and ERRα, plays a crucial role in regulating mitochondrial function, significantly contributing to the exercise capacity of skeletal muscle, so much so that targeting these factors has been proposed as a strategy to counteract aging and senescence (Vernier and Giguère, 2021). In fact, during differentiation of primary myocytes and C2C12, PGC-1α and ERRα transcripts are co-ordinately up-regulated shortly after myoblasts exit the cell cycle, which coincides with the onset of mitochondrial biogenesis (Murray and Huss, 2011; Huss et al., 2015).

Noteworthy, ERRα appears to be required for mitochondrial biogenesis in adult skeletal muscle and during muscle regeneration, suggesting its role in the regulation of muscle adaptations in response to physiological and pathological stimuli (LaBarge et al., 2014). Indeed, deletion of ERRα in mouse models significantly impairs mitochondrial oxidative capacity, confirming its central role in the regulation of cellular energy metabolism. Furthermore, ERRα appears to participate in myoblast differentiation and myotube formation, as in primary myocytes isolated from the hindlimbs of ERR^−/−^ mice, the early transition from proliferating myoblasts to fused multinucleated myotubes was inhibited, suggesting a potential role for ERRα in myotube formation and muscle atrophy (Huss et al., 2015).

Importantly, a pan-agonist of ERRs, called SLU-PP-332, has been recently synthesized and appears to replicate the muscle responses induced by exercise, promoting mitochondrial biogenesis and function, as well as cellular respiration in the murine C2C12 muscle cell line (Billon et al., 2023). Furthermore, the administration of SLU-PP-332 to male C57BL6/J mice promoted, through ERRs activation, an increase in oxidative type IIa skeletal muscle fibers and improvements in endurance and exercise capacity (Billon et al., 2023). Therefore, the activation of ERRs could modulate the expression of key molecules involved in muscle responses to exercise, such as NOX4, FNDC5, SIRT1, and PGC-1α, which are known to be down-regulated during aging. Since the expression of these factors heavily depends on exercise, levels of physical activity in the elderly population could influence their expression and, consequently, muscle homeostasis. However, the expression patterns of these mediators in muscle cells of the elderly remain poorly characterized, highlighting the importance of further exploring their role in maintaining muscle mass with aging. Finally, the possibility of replicating exercise-induced muscle responses could promote muscle health by encouraging higher levels of physical activity and reducing sedentary behaviour and the risk of falls during aging. In this context, targeting ERRs with the SLU-PP-332 agonist could represent an innovative strategy not only to counteract age-related muscle depletion but also to address other musculoskeletal disorders, metabolic conditions such as obesity, and neurodegenerative diseases.

Based on this evidence, the aim of our study was to i) characterize the expression profile of NOX4, SIRT1, PGC-1α, ERRα, and FNDC5 in muscle tissue collected from elderly individuals, both active and inactive, undergoing hip arthroplasty, and ii) investigate the effects of ERRs activation, through the treatment of SLU-PP-332, in primary cell cultures isolated from inactive individuals, to evaluate its targeting as a potential strategy to counteract muscle mass loss associated with conditions that lead to a sedentary lifestyle.

## 2 Materials and methods

### 2.1 Participants

A total of 20 women participants were enrolled at the Department of Orthopaedics and Traumatology of the Policlinico Tor Vergata during the period from January 8, 2024, to June 10, 2024, and underwent hip arthroplasty for osteoarthritis. Based on self-reported physical activity levels, they were divided into two experimental groups: 10 subjects practicing physical activity (active) and 10 sedentary subjects (inactive). Specifically, active participants reported regular moderate physical activity, mainly fast walking or bicycling, for at least 30 min a day, three times a week. In contrast, inactive participants did not engage in any regular physical activity or did so in a sporadic and unstructured manner.

Some comorbidities such as systemic hypertension, tachycardia, asthma and dyslipidaemia were found in the active group. These patients were receiving regular drug therapy that included enalapril maleate and lercanidipine hydrochloride for the treatment of hypertension, bisoprolol for the treatment of tachycardia, beclomethasone dipropionate and formoterol fumarate dihydrate for the treatment of asthma, and atorvastatin for the reduction of blood lipid levels. On the other hand, comorbidities such as systemic hypertension, tachycardia, hypercholesterolemia, diverticulosis, asthma, hepatic steatosis, and rhinitis were found in the inactive group. These patients regularly took enalapril maleate and lercanidipine hydrochloride, bisoprolol, atorvastatin, beclomethasone dipropionate, formoterol fumarate dihydrate, and oxatomide for the treatment of rhinitis. In addition, some patients in both groups reported taking omeprazole, as well as non-steroidal anti-inflammatory drugs (NSAIDs) and paracetamol sporadically.

Exclusion criteria included subjects with chronic viral infections, myopathies or other neuromuscular diseases, endocrine disorders of bone metabolism, diabetes or cancer, as well as subjects chronically receiving corticosteroids for autoimmune diseases or previous orthopaedic surgical implants.

### 2.2 Clinical evaluation

All participants underwent dual-energy X-ray absorptiometry (DXA) using a Lunar DXA apparatus (GE Healthcare, Madison, WI, United States). Scans of the lumbar spine (L1-L4) and femur (neck and total) were performed to measure the bone mineral density (BMD) in grams per square centimetre with a coefficient of variation of 0.7%, as indicated by the manufacturer. Measurements were taken 1 day before surgery on the non-dominant side, with the participants supine on an examination table with their limbs slightly abducted, and the results were expressed as *T*-scores. In addition, radiographic investigations were performed for the evaluation of hip osteoarthritis, using the radiographic atlas of Kellgren and Lawrence (K-L). The radiographs were reviewed independently by two orthopaedists, who considered all participants with a K-L ≥ 2 to be osteoarthritic (Canzone et al., 2024).

A pain assessment using visual analogue scale (VAS) scores (0–100 mm) was conducted for each participant prior to surgery, in association with the harris hip score (HHS) measurement for hip function. Finally, the handgrip strength test was performed using a hand-held dynamometer to measure the maximum isometric force exerted by the forearm muscles.

### 2.3 Specimen collection

A muscle biopsy sample from the superior portion of the vastus lateralis was taken for each participant during hip arthroplasty surgery. The samples were subsequently processed for qualitative and quantitative investigations according to the World Medical Association’s Code of Ethics (Declaration of Helsinki). The experimental procedures were approved by the Territorial Ethics Committee (CET) of Lazio Area 2 (approval reference number #25/23), and written informed consent was obtained from each participant prior to surgery.

### 2.4 Histological and morphometric analysis

Muscle biopsies taken from each participant were immediately fixed in 4% paraformaldehyde for 24 h and embedded in paraffin. For histological analysis, 3 μm thick sections were stained with hematoxylin and eosin (H&E) (Bio-Optica, Milan, Italy). A Nikon upright microscope ECLIPSE Ci-S (Nikon Corporation, Tokyo, Japan) connected to a Nikon digital camera was used to view the slides, while NIS-Elements software (5.30.01; Laboratory Imaging, Prague, Czech Republic) enabled images to be acquired at 40× magnification.

Two blind observers conducted the morphometric analysis by measuring the diameter of the muscle fibers. Specifically, measurements were taken at 40× magnification, resulting in a total of 3 non-overlapping readings for each participant, and shown as a mean ± standard error. A reference area was set using the NIS-Elements software, so that the dimensions of the region of interest were the same at each evaluation.

### 2.5 Immunohistochemistry

The expressions of NOX4, SIRT1, PGC-1α, ERRα and FNDC5 were assessed in muscle tissue by immunohistochemical analysis. Specifically, 3 μm-thick sections were pre-treated with ethylenediaminetetraacetic acid (EDTA) citrate, pH 6.0 for 20 min at 95 C and then incubated for 1 h with rabbit polyclonal anti-NOX4 antibody (dilution 1:100; BS6796, Bioworld Technology, Inc., United States), mouse monoclonal anti-SIRT1 antibody (dilution 1:100; ab110304, AbCam, Cambridge, United Kingdom), rabbit polyclonal anti-PGC-1α antibody (dilution 1:100; A87835, antibodies.com, Stockholm, Sweden), rabbit polyclonal anti-ERRα antibody (dilution 1:100; A90033, antibodies.com, Stockholm, Sweden), or rabbit polyclonal anti-FNDC5 C-terminal (dilution 1:100; ab181884, AbCam, Cambridge, United Kingdom). Washings were performed with phosphate buffered saline (PBS)/Tween20 (pH 7.6) (UCS Diagnostic, Rome, Italy). The horseradish peroxidase (HRP)-3,3′ diaminobenzidine (DAB) detection kit (UCS Diagnostic, Rome, Italy) was used to reveal immunohistochemical reactions. Specifically, 50 μL of DAB/450 μL of substrate were incubated for 3 min. The immunostaining background was evaluated with negative controls for each reaction, incubating the sections with secondary antibodies only (HRP) or with the detection system only (DAB) (Supplementary Figure S1).

The expression levels of NOX4, SIRT1, PGC-1α, ERRα and FNDC5 were assessed by mean optical density (MOD), a semiquantitative technique that estimates signal intensity in relation to the amount of protein present in tissue sections. Images were analyzed using NIS-Elements software (5.30.01; Laboratory Imaging, Prague, Czech Republic), which allowed identification of areas of interest containing chromogenic signal. MOD was calculated by measuring the signal intensity in these areas, providing a relative estimate of protein concentration for each sample analyzed. For each condition, the experiment was conducted in duplicate (n = 10 from N = 5 experiments).

### 2.6 Isolation and differentiation of primary cultures of myoblasts

Primary cultures of myoblasts were set up from muscle biopsies of active and inactive participants taken during hip arthroplasty surgery. Specifically, tissue samples were washed in PBS, fragmented into small portions using scissors, and then transferred to a falcon tube with PBS to be centrifuged at 340 rcf for 30 s. After removal of the supernatant, the fragments were subjected to enzymatic digestion with 2.5 mg/mL collagenase NB 4G Proved grade ≥0.18 U/mg (SERVA Electrophoresis GmbH, Heidelberg, DE) diluted in Dulbecco’s modified eagle medium (DMEM) F12 medium (MS01801009, Biowest, Nuaillé, France) and incubated at 37°C for 1 h under agitation. At the end of digestion, the supernatant was collected and centrifuged at 340 rcf for 10 min. The resulting pellet was resuspended in complete growth medium, consisting of Ham’s F14 (L0138, Biowest, Nuaillé, France) supplemented with 15% fetal bovine serum (FBS) (Biowest SAS, Nuaillé, France), 1 mg/mL insulin (Sigma-Aldrich, St. Louis, MO, United States), 2 mmol/L stable glutamine (Biowest SAS, Nuaillé, France), 100 Units/mL penicillin and 100 μg/mL streptomycin (Sigma-Aldrich, St. Louis, MO, United States), 5 μg/mL fibroblast growth factor (FGF) (SRP4037, Sigma-Aldrich, St. Louis, MO, United States) and 10 μg/mL epidermal growth factor (EGF) (SRP3027, Sigma-Aldrich, St. Louis, MO, United States). The cell suspension was then filtered and again centrifuged at 340 rcf for 10 min. Then, the cells were resuspended in complete culture medium and kept in a 37°C incubator with 5% CO_2_ until 80% confluence was reached, making medium changes every 2–3 days. After reaching enough cells of about 5 million, specific selection of myoblasts was performed using the anti-CD56 antibody (EasySep™ Human CD56 Positive Selection Kit II, Stemcell Technologies, Vancouver, Canada). Finally, the selected myoblasts were seeded in 24-well plates at a density of 2 × 10^4^ cells/well and maintained in complete culture medium in a 37°C incubator with 5% CO_2_, changing the medium every 2–3 days until confluence was reached. Differentiation into myotubes was induced at 90% confluence of primary myoblast cultures by adding the same culture medium without growth factors and with 5% FBS. The cells were maintained in culture medium in an incubator at 37°C with 5% CO_2_, changing the medium every 2 days for 15 days.

### 2.7 Primary cell cultures conditioned with SLU-PP-332

Primary cell cultures were treated with SLU-PP-332 to determine its effects on muscle metabolism or differentiation process. Specifically, first or second passage cells were seeded in a 24-well plate at a density of 2 × 10^4^ cells/well and treated with SLU-PP-332 at the concentration of 1 × 10^−5^ M for 48 h. All treated cell cultures were subjected to the same experimental procedures as untreated cells.

### 2.8 Immunofluorescence

The expression of paired box 7 (Pax7) and myoblast determination protein 1 (MyoD) was investigated to characterize primary cultures of myoblasts, as well as the expression of ERRα to study its effects in muscle metabolism, in primary cultures of myoblasts by immunofluorescence analysis. In addition, myosin heavy chain (MyHC) expression was evaluated to verify the success of the differentiation process in myotubes. Briefly, after fixation in 4% paraformaldehyde dissolved in 0.9% saline solution for 30  min, cell cultures were pretreated with EDTA citrate, pH 7.8 for 20 min at 95 C, and incubated for 1 h with rabbit polyclonal anti-Pax7 antibody (dilution 1:100; ab187339, AbCam, Cambridge, United Kingdom), mouse monoclonal anti-MyoD antibody (dilution 1:100; InvitrogenTM, ThermoFisher Scientific, United States), rabbit polyclonal anti-ERRα antibody (dilution 1:100; A90033, antibodies.com, Stockholm, Sweden), or mouse monoclonal anti-MyHC antibody (diluition 1:100; ab51263, AbCam, Cambridge, United Kingdom). Reaction was revealed by using secondary antibodies (dilution 1:1,000; A-11004, A-11008, Alexa Fluor® 488, Thermo Fisher Scientific, Waltham, MA United States). Washing was performed with PBS/Tween20 pH 7.6 (UCS Diagnostic, Rome, Italy). Finally, samples were counteracted with 4′,6-diamidino-2-phenylindole (DAPI) counterstain (Kreatech Biotechnology B.V., Amsterdam, Netherlands).

A Nikon upright microscope ECLIPSE Ci-S (Nikon Corporation, Tokyo, Japan) connected to a Nikon digital camera was used to view the images, while the NIS-Elements software (5.30.01; Laboratory Imaging, Prague, Czech Republic) was used to capture them at 20× magnification.

### 2.9 Cell viability assessment

CellTiter 96 AQueous One (Promega, Madison, WI, United States) was used to identify viable cells. This colorimetric method incorporates a tetrazolium compound (3-(4,5-dimethylthiazol-2-yl)-5-(3-carboxymethoxyphenyl)-2-(4-sulphophenyl)-2H-tetrazolium-MTS) and an electron coupling reagent (phenazinamethosulphate-PMS). As previously described (Cariati et al., 2023), the conversion of MTS to soluble formazan in the culture medium generates a dye whose absorbance was measured using a microplate reader (Spark Multimode Microplate Reader-Tecan, Austria). The absorbance provides a measure of cell viability and allows the identification of any toxicity point of the administered substance. For each condition, the experiment was conducted in triplicate (n = 9 from N = 3 experiments).

### 2.10 Measurement of lactate dehydrogenase (LDH) cytotoxicity

The LDH-GloTM Cytotoxicity Assay (J2380, Promega, Madison, Unites States) was used to measure cytotoxicity by detecting and quantifying lactate dehydrogenase (LDH) levels in the supernatant. As instructed by the manufacturer, LDH detection reagent, containing lactate, NAD^+^, reductase, reductase substrate and Ultra-GloTM rLuciferase, was added to a diluted cell culture medium sample. Specifically, cells were plated at a density of 5 × 10^3^ in a 96-well plate. At the end of treatment, 4 μL of culture medium was taken from each well and added to 46 μL of LDH storage buffer (TRIS-HCl 200 mM pH 7.3, 10% glycerol and 1% BSA) in an empty 96-well plate. 50 μL of LDH detection reagent (50 μL LDH detection enzyme mixture + 0.25 μL reductase substrate) was added to each well, reaching the 25× dilution suggested by the manufacturer, and incubated for 60 min at 37°C. The luminescent signal, proportional to the amount of LDH present in the sample, was measured with a microplate reader (Spark Multimode Microplate Reader-Tecan, Austria). For each condition, the experiment was conducted in quintuplicate (n = 25 from N = 5 experiments).

### 2.11 Measurement of intracellular ROS level

Intracellular ROS levels were measured using the fluorescent probe 2‘,7’-dichlorodihydrofluorescein diacetate (H2DCFDA) (D399, InvitrogenTM, ThermoFisher Scientific, United States). As previously described (Bonanni et al., 2024), cell samples were washed with PBS and incubated with 10 μM H2DCFDA for 40 min at 37°C in the dark. The mean fluorescence intensity was measured using a microplate reader (Spark Multimode Microplate Reader-Tecan, Austria). For each condition, the experiment was conducted in quintuplicate (n = 25 from N = 5 experiments).

### 2.12 Reduced glutathione (GSH) quantification

GSH levels were quantified in primary cultures of myoblasts using the GSH-Glo™ assay (V6911, Promega, Madison, United States). Briefly, cells were plated at a density of 5 × 10^3^ in a 96-well plate and cultured according to experimental procedures. At the end of treatment, the culture medium was removed, and a wash in 1X PBS was performed. 100 μL of GSH-Glo™ 1X reagent containing Luciferin-NT substrate and Glutathione S-Transferase diluted 1:100 in GSH-Glo™ reaction buffer was added to each well. The plate was mixed briefly on a shaker and incubated at room temperature for 30 min. Next, 100 µL of reconstituted luciferin detection reagent was added to each well, followed by brief mixing and an additional 15-min incubation. GSH levels were measured as luminescence using a microplate reader (Spark Multimode Microplate Reader-Tecan, Austria), and expressed as counts per second with an integration time of one second (Jacobsen et al., 2018). For each condition, the experiment was conducted in quintuplicate (n = 25 from N = 5 experiments).

### 2.13 Senescence β-galactosidase activity (SA-β-gal) assay

SA-β-gal was quantified in primary cultures of myoblasts using the SA-β-gal assay kit (23,833, Cell Signaling Technology, Inc., Danvers, MA, United States). For protein extraction, 1X senescence cell lysis buffer was prepared with 1.0 mM (phenylmethanesulfonylfluoride) PMSF and a protease/phosphatase inhibitor cocktail. After removing the culture medium, cells were washed with 1X PBS, lysed with 100 μL of cold 1X senescence cell lysis buffer, incubated on ice for 5 min and harvested by scraping. The lysate was homogenized and centrifuged at 14,000 rpm for 5 min at 4°C. Meanwhile, the 2X assay buffer was prepared by adding 10 mM β-mercaptoethanol to 2X senescence reaction buffer, and SA-β-gal substrate was diluted to 1X with the same buffer. Then, 50 μL of cell lysate and 50 μL of 2X reaction buffer were mixed in a 96-well plate and incubated at 37°C, protected from light, for 1–3 h. Finally, 50 μL of the reaction mixture was transferred to a black opaque 96-well plate, and the reaction was blocked with 200 μL of senescence arresting solution per well. Fluorescence was measured at 360 nm excitation and 465 nm emission using a microplate reader (Spark Multimode Microplate Reader-Tecan, Austria). For each condition, the experiment was conducted in quintuplicate (n = 25 from N = 5 experiments).

### 2.14 Western blotting analysis

A western blotting analysis was performed to investigate the expression of NOX4, SIRT1, PGC-1α, ERRα, FNDC5, Akt and B-cell lymphoma 2 (Bcl-2) in muscle tissue and primary cultures of myoblasts derived from experimental groups. Proteins samples extracted by using RIPA buffer were separated by 8%–16% precast SDS-PAGE (Bio-Rad, Hercules, CA, United States) under reduced conditions. Protein concentration was determined using the Pierce BCA Protein Assay Kit (Thermo Scientific, Vilnius, Lithuania). Equal amounts of protein (20 μg) were resolved on 8%–16% precast SDS-PAGE and transferred to PVDF membrane. Then membranes were incubated with rabbit polyclonal anti-NOX4 antibody (dilution 1:1,000; BS6796, Bioworld Technology, Inc., United States), mouse monoclonal anti-SIRT1 antibody (dilution 1:1,000; ab110304, AbCam, Cambridge, United Kingdom), rabbit polyclonal anti-PGC-1α antibody (dilution 1:1,000; A87835, antibodies.com, Stockholm, Sweden), rabbit polyclonal anti-ERRα antibody (dilution 1:1,000; A90033, antibodies.com, Stockholm, Sweden), rabbit polyclonal anti-FNDC5 C-terminal (dilution 1:1,000; ab181884, AbCam, Cambridge, United Kingdom), rabbit monoclonal anti-Akt antibody (dilution 1:1,000; #4685 Cell Signalling Technology, Massachusetts, United States), or mouse monoclonal anti-Bcl-2 antibody (dilution 1:1,000; #15071 Cell Signalling Technology, Massachusetts, United States) and successively with anti-rabbit IgG coupled to HRP or anti-mouse IgG coupled to HRP, respectively. Moreover, the same membranes were incubated with mouse monoclonal anti-GAPDH (dilution 1:5,000; ab8245, AbCam, Cambridge, United Kingdom) used for normalization. Immunoreactive electrophoretic bands were detected by enhanced chemiluminescence (ECL Advance, Amersham; GE Healthcare Life Sciences, Little Chalfont, Buckinghamshire, United Kingdom) using a VersaDoc 5,000 Imager (Bio-Rad).

The expression levels of NOX4, SIRT1, PGC-1α, ERRα, FNDC5, Akt and Bcl-2 were quantified by calculating the densitometric values of the relevant bands and normalizing the results against the GAPDH expression, expressing them as mean ± standard error. The original western blotting images are shown in Supplementary Figures S2 and S3.

### 2.15 Statistical analysis

All statistical analyses were conducted using GraphPad Prism 8 software (GraphPad Prism 8.0.1, La Jolla, CA, Unites States). All data with a normal distribution were processed with Welch’s parametric test and correlations were conducted with Spearman’s correlation analysis. Western blotting data on primary myoblast cultures were compared using one-way ANOVA and Tukey’s multiple comparison test. All data were expressed as mean ± standard error and considered significantly different if p < 0.05.

## 3 Results

### 3.1 Clinical evaluation

Our study involved 20 women undergoing hip arthroplasty for coxarthrosis who were divided into two experimental groups based on self-reported physical activity levels: 10 subjects who practiced physical activity (active group) and 10 sedentary subjects (inactive group).

As shown in Table 1, clinical and instrumental evaluation was performed to analyze several parameters for each participant, including age (years), *T*-score (L1-L4), *T*-score (femoral neck), *T*-score (total femur), body mass index (BMI) (Kg/m^2^), as well as serum levels of 25-(OH)-Vit D (ng/mL) and parathyroid hormone (PTH) (pg/mL), and VAS scores. In addition, HHS and Handgrip strength (kg) were assessed to measure hip function and maximum isometric force exerted by forearm muscles, respectively.

**TABLE 1:** Study population characteristics.

Parameters	Active (n = 10)	Inactive (n = 10)	Significance
Age (years)	77.6 ± 1.2	78.9 ± 1.1	p = 0.43
*T*-score (L1–L4)	0.5 ± 0.4	−1.8 ± 0.4	p < 0.01
*T*-score (femoral neck)	0.2 ± 0.5	−1.9 ± 0.3	p < 0.01
*T*-score (total femur)	0.1 ± 0.6	−2.4 ± 0.2	p < 0.01
BMI (Kg/m^2^)	23.8 ± 0.8	29.3 ± 1.3	p < 0.05
25-(OH)-Vit D (ng/mL)	23.9 ± 2.9	14.7 ± 1.6	p < 0.05
PTH (pg/mL)	100.6 ± 11.1	110.5 ± 13.5	p = 0.58
VAS	5.5 ± 0.3	8.2 ± 0.4	p < 0.0001
HHS	54.5 ± 4.2	21.7 ± 2.5	p < 0.0001
Handgrip strength (kg)	20.1 ± 0.8	13.8 ± 0.8	p < 0.0001

BMI, body mass index; PTH, parathyroid hormone; VAS, visual analogue scale; HHS, harris hip score.

First, there were no discrepancies by age between active (77.6 ± 1.2) and inactive (78.9 ± 1.1) subjects (p = 0.43). In contrast, BMD assessment of the lumbar spine, femoral neck and total femur, expressed as *T*-score, showed statistically significant differences between the two groups (p < 0.01). Specifically, the active group was characterized by *T*-score (L1-L4), *T*-score (femoral neck) and *T*-score (total femur) values of 0.5 ± 0.4, 0.2 ± 0.5 and 0.1 ± 0.6, respectively; while values of −1.8 ± 0.4, −1.9 ± 0.3 and −2.4 ± 0.2 were found for *T*-score (L1-L4), *T*-score (femoral neck) and *T*-score (total femur), respectively, in the inactive group. Similarly, a statistically significant difference was found between the two experimental groups for BMI (active group: 23.8 ± 0.8; inactive group: 29.3 ± 1.3; p < 0.05) and serum levels of 25-(OH)-Vit D (active group: 23.9 ± 2.9; inactive group: 14.7 ± 1.6; p < 0.05). On the other hand, similar serum PTH levels were measured between active and inactive subjects (active group: 100.6 ± 11.1; inactive group: 110.5 ± 13.5; p = 0.58).

Finally, a statistically significant difference for VAS, HHS and Handgrip strength scores was found between the two experimental groups (p < 0.0001). In fact, VAS scores were 5.5 ± 0.3 in the active group and 8.2 ± 0.4 in the inactive group. In agreement, marked impairment of hip function and reduced muscle strength measurement were found in the sedentary subjects, characterized by HHS of 21.7 ± 2.5 and Handgrip strength values of 13.8 ± 0.8. In contrast, the active group had HHS of 54.5 ± 4.2 and Handgrip strength values of 20.1 ± 0.8.

### 3.2 Correlation analysis between perceived pain, hip function, and muscle strength

A Spearman correlation analysis was conducted to investigate possible relationships between perceived pain, hip function, and muscle strength of all study participants.

**FIGURE 1:**
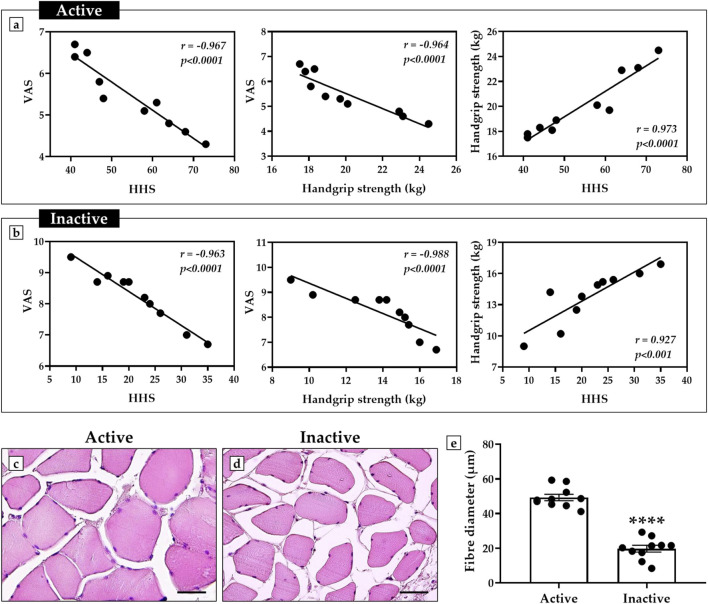
Correlation analysis of perceived pain, hip function, and muscle strength in active and inactive subjects. **(a)** Active group: negative correlation between visual analogue scale (VAS) scores and harris hip score (HHS) (n = 10, r = −0.967, p < 0.0001); negative correlation between VAS scores and handgrip strength (n = 10, r = −0.964, p < 0.0001); positive correlation between handgrip strength and HHS (n = 10, r = 0.973, p < 0.0001). **(b)** Inactive group: negative correlation between VAS scores and HHS (n = 10, r = −0.963, p < 0.0001); negative correlation between VAS scores and handgrip strength (n = 10, r = −0.988, p < 0.0001); positive correlation between handgrip strength and HHS (n = 10, r = 0.927, p < 0.001). **(c–e)** Hematoxylin and eosin (H&E)-stained sections of muscle tissue from active and inactive subjects. The graph shows a significant reduction in muscle fiber diameter in sedentary subjects compared to the active group (p < 0.0001). For each patient, muscle fibre diameter measurements were taken in triplicate (n = 30 from N = 10 patients) and shown as a mean ± error standard. For 40× images, scale bar represents 50 μm.

Figures 1a,b shows a negative correlation between VAS and HHS scores in both active and inactive subjects, since as pain increases, hip function decreases. Similarly, VAS scores were negatively correlated with Handgrip strength measures, as evidenced by increasing pain to decreasing muscle strength. In contrast, a positive correlation was observed between Handgrip strength and HHS measures, as greater muscle strength was found to be associated with better hip function.

In agreement, histological and morphometric analysis showed impaired muscle quality in the inactive subjects, characterized by smaller fiber diameter compared with the muscle of active subjects (Figures 1c,d). In fact, the mean value of muscle fiber diameter was 49.3 ± 1.8 in the active group and 19.8 ± 1.9 in the inactive group (p < 0.0001) (Figure 1e).

### 3.3 Differential expression of NOX4, SIRT1, PGC-1α, ERRα and FNDC5 in muscle tissue of active and inactive subjects

An immunohistochemistry analysis was conducted to investigate possible variations in the expression pattern of NOX4, SIRT1, PGC-1α, ERRα, and FNDC5 in muscle tissue of active and inactive subjects, using MOD as the measurement value to indicate the expression levels of these proteins.

**FIGURE 2:**
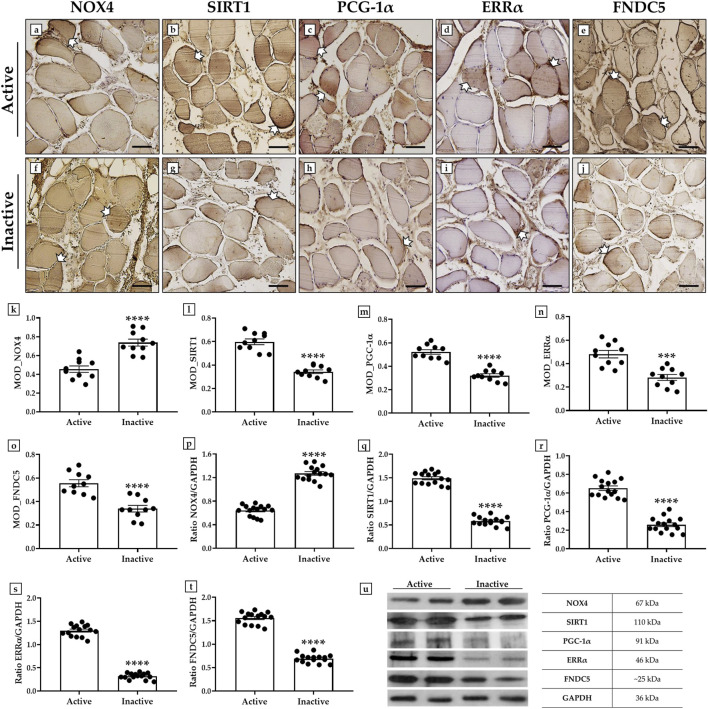
Evaluation of NADPH oxidase 4 (NOX4), sirtuin 1 (SIRT1), peroxisome proliferator-activated receptor gamma coactivator 1-alpha (PGC-1α), estrogen related receptor alpha (ERRα) and fibronectin type III domain-containing protein 5 (FNDC5) expression in muscle tissue of active and inactive subjects by immunohistochemistry and western blotting analysis. **(a, f, k)** White arrows indicate representative NOX4-positive areas in the muscle tissue. The highest MOD values for NOX4 were found in the muscle of the inactive group respect to the active group (p < 0.0001). **(b, g, l)** White arrows indicate representative SIRT1-positive areas in the muscle tissue. The highest MOD values for SIRT1 were found in the muscle of the active group respect to the inactive group (p < 0.0001). **(c, h, m)** White arrows indicate representative PGC-1α-positive areas in the muscle tissue. The highest MOD values for PGC-1α were found in the muscle of the active group respect to the inactive group (p < 0.0001). **(d, i, n)** White arrows indicate representative ERRα-positive areas in the muscle tissue. The highest MOD values for ERRα were found in the muscle of the active group respect to the inactive group (p < 0.001). **(e, j, o)** White arrows indicate representative FNDC5-positive areas in the muscle tissue. The highest MOD values for FNDC5 were found in the muscle of the active group respect to the inactive group (p < 0.0001). For 20× images, scale bar represents 100 μm (n = 10 from N = 5 experiments). **(p, u)** The higher expression of NOX4 was measured in muscle tissue of inactive group compared to the active subjects (p < 0.0001). **(q, u)** The higher expression of SIRT1 was measured in muscle tissue of active group compared to the inactive subjects (p < 0.0001). **(r, u)** The higher expression of PGC-1α was measured in muscle tissue of active group compared to the inactive subjects (p < 0.0001). **(s, u)** The higher expression of ERRα was measured in muscle tissue of active group compared to the inactive subjects (p < 0.0001). **(t, u)** The higher expression of FNDC5 was measured in muscle tissue of active group compared to the inactive subjects (p < 0.0001). For each condition, the experiment was conducted in triplicate (n = 15 from N = 5 experiments).

Importantly, a significant increase in NOX4, the main inducer of oxidative stress and marker of muscle atrophy, was observed in sedentary subjects, with MOD values of 0.45 ± 0.03 in the active group and 0.74 ± 0.04 in the inactive group (p < 0.0001) (Figures 2a,f,k). In contrast, the levels of SIRT1, PGC-1α, ERRα and FNDC5, all promoters of mitochondrial biogenesis, were significantly increased in the muscle tissue of the active group. In detail, the MOD values for SIRT1 were 0.59 ± 0.02 in the active group and 0.34 ± 0.02 in the inactive group (p < 0.0001) (Figures 2b,g,l). Similarly, the MOD values for PGC-1α were 0.52 ± 0.02 in the active group and 0.32 ± 0.02 in the inactive group (p < 0.0001) (Figures 2c,h,m); whereas, the MOD values for ERRα were 0.48 ± 0.03 in the active group and 0.28 ± 0.03 in the inactive group (p < 0.001) (Figures 2d,i,n). Finally, the MOD values for FNDC5 were 0.56 ± 0.03 in the active group and 0.34 ± 0.03 in the inactive group (p < 0.0001) (Figures 2e,j,o).

The results of immunohistochemistry were confirmed by wester blotting analysis, which showed a positive band at about 67 kDa, corresponding to the molecular weight of NOX4, a positive band at about 110 kDa, corresponding to the molecular weight of SIRT1, a positive band at about 91 kDa, corresponding to the molecular weight of PGC-1α, a positive band at about 46 kDa, corresponding to the molecular weight of ERRα, and a positive band at about 25 kDa, corresponding to the molecular weight of FNDC5, in the protein extracts of all muscle tissue samples. Particularly, Figures 2p–u shows that the highest expression of NOX4 was measured in the inactive subjects; while SIRT1, PGC-1α, ERRα and FNDC5 were more highly expressed in the muscle tissue of the active group. In fact, the mean values of NOX4 expression obtained by densitometric analysis were 0.64 ± 0.06 in the active group and 1.27 ± 0.08 in the inactive group (p < 0.0001) (Figures 2p,u). In contrast, the mean expression values of SIRT1 were 1.49 ± 0.08 in the active group and 0.58 ± 0.06 in the inactive group (p < 0.0001) (Figures 2q,u); while, the mean expression values of PGC-1α were 0.65 ± 0.06 in the active group and 0.26 ± 0.05 in the inactive group (p < 0.0001) (Figures 2r,u). In agreement, the mean values of ERRα expression were 1.30 ± 0.06 in the active group and 0.32 ± 0.04 in the inactive group (p < 0.0001) (Figures 2s,u). Finally, the mean expression values of FNDC5 were 1.56 ± 0.06 in the active group and 0.69 ± 0.06 in the inactive group (p < 0.0001) (Figures 2t,u).

### 3.4 Effects of SLU-PP-332 treatment on myoblasts isolated from skeletal muscle of inactive subjects

Primary cultures of myoblasts derived from inactive subjects were treated for 48 h with SLU-PP-332, a known agonist of ERRα, investigating its effects in terms of cell viability, LDH activity, intracellular ROS levels, GSH levels, and SA-β-gal.

**FIGURE 3:**
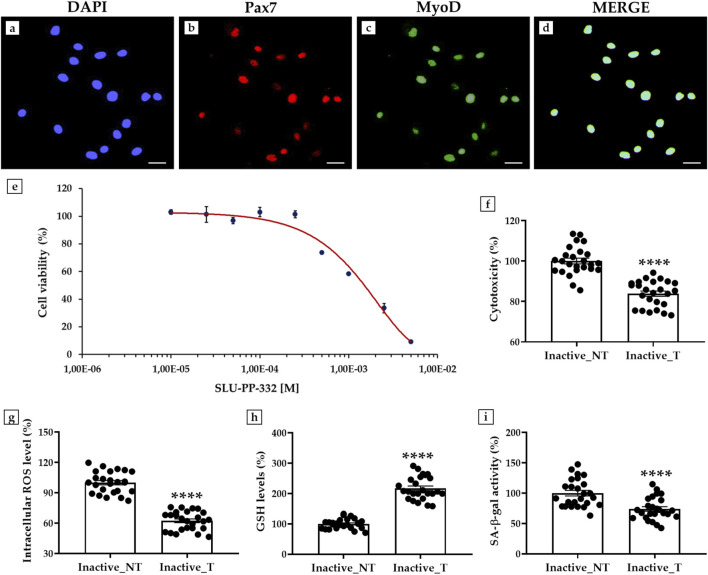
Effects of SLU-PP-332 treatment in myoblasts from inactive subjects on cell viability, cytotoxicity, oxidative stress, and senescence β-galactosidase activity (SA- β-gal). **(a–d)**: Immunofluorescence for Pax7 and MyoD in myoblasts: **(a)** Nuclei are stained with DAPI (blue); **(b)** Immunostaining for Pax7 (red); **(c)** Immunostaining for MyoD (green); **(d)** Merge for Pax7 and MyoD signals. 40× images, scale bar represents 100 μm. **(e)** MTS assay: the half inhibitory concentration (IC50) was obtained at a dosage between 1 × 10^−3^ M and 2.5 × 10^−3^ M (n = 9 from N = 3 experiments). **(f)** Lactate dehydrogenase (LDH) cytotoxicity assay: significant reduction of 16.1% of cell damage in SLU-PP-332-treated myoblasts (Inactive_T) compared with untreated cells (Inactive_NT) (p < 0.0001) (n = 25 from N = 5 experiments). **(g)** Intracellular reactive oxygen species (ROS) levels: significant reduction of 37.7% of oxidative stress in SLU-PP-332-treated myoblasts (Inactive_T) compared with untreated cells (Inactive_NT) (p < 0.0001) (n = 25 from N = 5 experiments). **(h)** Reduced glutathione (GSH) assay: significant increase of 117.4% in intracellular GSH levels in SLU-PP-332-treated myoblasts (Inactive_T) compared with untreated cells (Inactive_NT) (p < 0.0001) (n = 25 from N = 5 experiments). **(i)** SA-β-gal assay: significant reduction of 26.1% of enzymatic activity in SLU-PP-332-treated myoblasts (Inactive_T) compared with untreated cells (Inactive_NT) (p < 0.0001) (n = 25 from N = 5 experiments).

First, an immunofluorescence analysis was performed to characterize myoblasts by assessing the expression of Pax7, a specific marker of quiescent and activated satellite cells, and MyoD, a transcription factor involved in myogenesis and muscle differentiation (Le Grand and Rudnicki, 2007) (Figures 3a–d). Fluorescent signal analysis showed clear co-expression of Pax7 and MyoD (Figure 3d), confirming the proliferative and differentiative abilities of these cells.

Next, a dose-response curve was constructed to estimate the doses of SLU-PP-332 for which nontoxic effects were detected by treating cells with increasing concentrations of the substance and then performing an MTS assay to measure the population of viable cells. As shown in Figure 3e, cell viability was not affected up to a dosage of 1 × 10^−4^ M, while a progressive reduction in the percentage of viable cells was detected as the concentration of the substance increased. Furthermore, half of the inhibitory concentration (IC50) was obtained at a dosage between 1 × 10^−3^ M and 2.5 × 10^−3^ M. Therefore, myoblast cultures were treated with SLU-PP-332 at a concentration of 1 × 10^−5^ M.

Interestingly, the LDH activity assay showed a significant reduction in cell damage in myoblasts treated with SLU-PP-332. In fact, the percentage of cytotoxicity was 100.0 ± 1.4 in untreated cells (Inactive_NT); while it decreased to 83.9 ± 1.3 in treated cells (Inactive_T), corresponding to a reduction of 16.1% (p < 0.0001) (Figure 3f).

In agreement, measurement of intracellular ROS levels showed a significant reduction in oxidative stress, with values of 100.0 ± 2.1 in the Inactive_NT group and 62.3 ± 1.9 in the Inactive_T group (p < 0.0001). In this case, the relative reduction in intracellular ROS levels was 37.7% (Figure 3g).

In addition, SLU-PP-332 treatment promoted a 117.4% increase in intracellular GSH levels in myoblasts from inactive subjects, indicating a substantial enhancement of cellular antioxidant capacity. In fact, GSH levels were 100.0 ± 3.3 in the Inactive_NT group and 217.4 ± 7.6 in the Inactive_T group (p < 0.0001) (Figure 3h).

Finally, a significant reduction in SA-β-gal was observed after treatment with the ERRα agonist, suggesting its modulating effect on cell aging. Specifically, enzyme activity decreased by 26.1%, from 100.0 ± 4.5 in the Inactive_NT group to 73.9 ± 3.7 in the Inactive_T group (p < 0.0001) (Figure 3i).

### 3.5 Evaluation of NOX4, SIRT1, PGC-1α, ERRα, FNDC5, Akt and Bcl-2 expressions in myoblasts treated with SLU-PP-332

A western blotting analysis was conducted to investigate the expression of NOX4, SIRT1, PGC-1α, ERRα, and FNDC5 in primary cultures of myoblasts derived from muscle biopsies of inactive subjects after SLU-PP-332 treatment (Inactive_T) compared to untreated inactive subjects (Inactive_NT) and untreated active subjects (Active_NT). In addition, the expression of Akt and Bcl-2 was measured to investigate possible changes in cell proliferation and survival processes.

**FIGURE 4:**
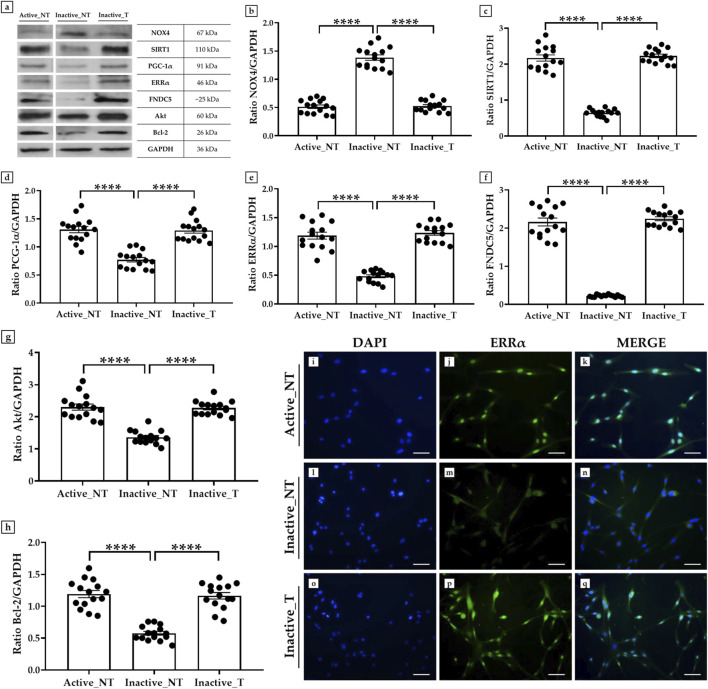
Evaluation of the expression of NADPH oxidase 4 (NOX4), sirtuin 1 (SIRT1), peroxisome proliferator-activated receptor gamma coactivator 1-alpha (PGC-1α), estrogen-related receptor alpha (ERRα), fibronectin type III domain-containing protein 5 (FNDC5), Akt and B-cell lymphoma 2 (Bcl-2) in primary cultures of myoblasts from active and inactive subjects by western blotting and immunofluorescence analysis. **(a–h)** Western blotting analysis: **(a, b)** The highest expression of NOX4 was measured in untreated cells from the inactive group (Inactive_NT) compared to untreated myoblasts from the active group (Active_NT) and treated myoblasts from the inactive group (Inactive_NT) (Active_NT vs. Inactive_NT and Inactive_NT vs. Inactive_T, p < 0.0001). SLU-PP-332 treatment promoted a significant increase in SIRT1 **(a, c)**, PGC-1α **(a, d)**, ERRα **(a, e)**, FNDC5 **(a, f)**, Akt **(a, g)**, and Bcl-2 **(a, h)** expressions in all cell samples, similar to the levels measured in the Active_NT group (Active_NT vs. Inactive_NT and Inactive_NT vs. Inactive_T, p < 0.0001). For each condition, the experiment was conducted in triplicate (n = 15 from N = 5 experiments). **(i–q)** Immunofluorescence analysis for ERRα in muscle cells from active and inactive subjects: **(i, l, o)** nuclei are stained with DAPI (blue); **(j, m, p)** immunostaining for ERRα (green); **(k, n, q)** merge for DAPI and ERRα signals. 20× images, scale bar represents 50 μm.

In agreement with the results on muscle tissue, western blotting analysis showed a positive band at about 67 kDa, corresponding to the molecular weight of NOX4, a positive band at about 110 kDa, corresponding to the molecular weight of SIRT1, a positive band at about 91 kDa, corresponding to the molecular weight of PGC-1α, a positive band at about 46 kDa, corresponding to the molecular weight of ERRα, a positive band at about 25 kDa, corresponding to the molecular weight of FNDC5, a positive band at about 60 kDa, corresponding to the molecular weight of Akt, and a positive band at about 26 kDa, corresponding to the molecular weight of Bcl-2, in the protein extracts of all cellular samples (Figure 4a). Not surprisingly, NOX4 was more highly expressed in the absence of SLU-PP-332 treatment, with mean values of 0.51 ± 0.06 in the Active_NT group, 1.38 ± 0.12 in the Inactive_NT group and 0.53 ± 0.05 in the Inactive_T group (Active_NT vs. Inactive_NT and Inactive_NT vs. Inactive_T, p < 0.0001) (Figure 4b). In contrast, a significant increase in the expression of SIRT1, PGC-1α, ERRα and FNDC5 was detected in the treated cells of all inactive subjects, with values comparable to those detected in the untreated myoblasts of active subjects. In fact, the mean expression values of SIRT1 obtained by densitometric analysis were 2.17 ± 0.18 in the Active_NT group, 0.65 ± 0.06 in the Inactive_NT group and 2.23 ± 0.11 in the Inactive_T group (Active_NT vs. Inactive_NT and Inactive_NT vs. Inactive_T, p < 0.0001) (Figure 4c); whereas the mean expression values of PGC-1α were 1.31 ± 0.13 in the Active_NT group, 0.77 ± 0.09 in the Inactive_NT group and 1.29 ± 0.11 in the Inactive_T group (Active_NT vs. Inactive_NT and Inactive_NT vs. Inactive_T, p < 0.0001) (Figure 4d). Similarly, the mean expression values of ERRα were 1.19 ± 0.14 in the Active_NT group, 0.48 ± 0.06 in the Inactive_NT group and 1.23 ± 0.10 in the Inactive_T group (Active_NT vs. Inactive_NT and Inactive_NT vs. Inactive_T, p < 0.0001) (Figure 4e). Finally, the mean expression values of FNDC5 were 2.16 ± 0.25 in the Active_NT group, 0.22 ± 0.02 in the Inactive_NT group and 2.25 ± 0.13 in the Inactive_T group (Active_NT vs. Inactive_NT and Inactive_NT vs. Inactive_T, p < 0.0001) (Figure 4f).

Noteworthy, a positive modulation in the expression pattern of Akt and Bcl-2 was promoted by treatment with SLU-PP-332 in the muscle cells of inactive subjects, in line with the expression values measured in active subjects. In detail, densitometric analysis revealed mean expression values for Akt of 2.30 ± 0.18 in the Active_NT group, 1.36 ± 0.12 in the Inactive_NT group and 2.27 ± 0.13 in the Inactive_T group (Active_NT vs. Inactive_NT and Inactive_NT vs. Inactive_T, p < 0.0001) (Figure 4g); whereas mean expression values for Bcl-2 were 1.19 ± 0.12 in the Active_NT group, 0.58 ± 0.07 in the Inactive_NT group and 1.16 ± 0.12 in the Inactive_T group (Active_NT vs. Inactive_NT and Inactive_NT vs. Inactive_T, p < 0.0001) (Figure 4h).

Finally, an immunofluorescence analysis was conducted to investigate the expression of ERRα, whose interaction with the coactivator PGC-1α is known to be critical in the regulation of metabolic and energetic processes, such as mitochondrial biogenesis (Malik et al., 2023). Notably, cells in the Inactive_T group showed a marked fluorescent signal for ERRα (Figures 4o–q), like that detected in cells from the Active_NT group (Figures 4i–k). In contrast, the protein was only weakly expressed in the absence of treatment with SLU-PP-332 in cells from the Inactive_NT group (Figures 4l–n).

### 3.6 Impact of ERRs targeting on the differentiation process

**FIGURE 5:**
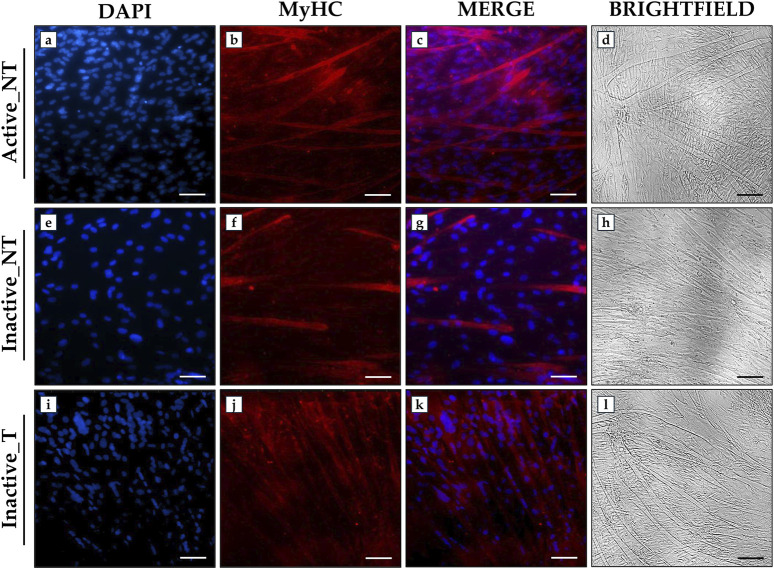
Analysis of myosin heavy chain (MyHC) expression in primary myotube cultures from active and inactive subjects using immunofluorescence analysis. **(a, e, i)** nuclei are stained with DAPI (blue); **(b, f, j)** immunostaining for MyHC (red); **(c, g, k)** merge for DAPI and MyHC signals. **(d, h, l)** Brightfield images of myotubes after 15 days of differentiation. 20× images, scale bar represents 50 μm.

An immunofluorescence analysis was performed to evaluate the effects of ERRs targeting on myotube formation by analyzing MyHC expression after 15 days of differentiation. Immunostaining for this marker, which is essential for the formation of multinucleated myotubes (Langendorf et al., 2020), showed marked differences between the experimental groups. Particularly, SLU-PP-332 treatment promoted an abundant fluorescent signal for MyHC and numerous myotube formation in the Inactive_T group (Figures 5i–k), comparable to that observed in the Active_NT group (Figures 5a–c). Conversely, a less marked fluorescent signal for MyHC was observed in the Inactive_NT group, associated with less myotube formation (Figures 5e–g). In agreement, brightfield imaging revealed abundant myotube formation in the Active_NT (Figure 5d) and Inactive_T (Figure 5l) groups compared to the Inactive_NT group (Figure 5h).

## 4 Discussion

Regular exercise profoundly influences musculoskeletal metabolism, improving its mass, structure, and homeostasis and helping to reduce the risk of frailty, disability, and mortality (Mølmen et al., 2025). In contrast, a sedentary lifestyle promotes, in late life, the development of a condition of muscle atrophy that predisposes to functional limitation and amplifies the algic symptoms associated with age-related musculoskeletal disorders (Zhang et al., 2025a). Therefore, our study analyzed the molecular profile of skeletal muscle in active and inactive individuals and evaluated the effects of ERRs activation by SLU-PP-332 in primary cells from inactive individuals to explore its role in counteracting sedentary-induced muscle loss.

### 4.1 Differences in VAS, HHS and handgrip strength scores between active and inactive subjects

A total of 20 elderly women were enrolled in this study and divided into an active group and an inactive group based on self-reported physical activity. Some comorbidities were detected in the participants, such as systemic hypertension, tachycardia, asthma, dyslipidaemia, hypercholesterolaemia, diverticulosis and hepatic steatosis, which required specific pharmacological treatments. These conditions, as well as the associated pharmacotherapy, could potentially influence musculoskeletal metabolism. However, it is important to note that the distribution of comorbidities found, and their pharmacological treatment was absolutely comparable between the two groups, suggesting that the experimental evidence reported here is attributable to self-reported levels of physical activity.

Our results showed a linear correlation between VAS scores, Handgrip strength and hip function. Specifically, active subjects were characterized by less functional limitation than the inactive group, in association with higher Handgrip strength measures and a significant reduction in VAS scores. These data agree with the observations of Long et al. who reported the existence of a correlation between Handgrip strength and HHS in 202 patients undergoing total hip arthroplasty surgery (Long et al., 2021), highlighting the influence of exercise in joint function. Moreover, the presence of a higher pain component in inactive subjects, correlated with Handgrip strength and HHS measurements, confirms the drastic contribution of musculoskeletal pain to functional limitation and sedentary behavior, predisposing to reduced muscle fiber diameter (Metcalfe et al., 2019; Lin et al., 2022; Chen et al., 2023). Fortunately, regular exercise represents an effective strategy to limit the influence of pain on joint functionality, reinforcing the importance of physical activity in the well-being of patients with age-related musculoskeletal disorders (Krauß et al., 2014; Sasaki et al., 2022; Marriott et al., 2024).

### 4.2 Comparison of NOX4, SIRT1, PGC-1α, ERRα, and FNDC5 expression in muscle tissue of active and inactive subjects

Clinical and instrumental assessment was confirmed by morphometric analysis, as evidenced by the reduced diameter of muscle fibers observed in inactive individuals, likely due to low levels of physical activity. In agreement, immunohistochemical and western blot analyses highlighted significant expression changes in a wide range of factors involved in muscle adaptations to exercise.

Among these, NOX4 was found to be more highly expressed in the muscle tissue of inactive women, suggesting its involvement in muscle atrophy characteristic of aging, as previously reported (Ferreira and Laitano, 2016). However, current knowledge about its impact on musculoskeletal metabolism and exercise responses remains conflicting (Kumar et al., 2023). In this regard, Deng et al. recently demonstrated that the administration of a non-steroidal anti-inflammatory drug to counteract diabetic sarcopenia in a mouse model promoted NOX4 downregulation and reduced ROS production (Deng et al., 2024). In agreement, Wu and colleagues administered Benzo[a]pyrene to the murine C2C12 cell line to reproduce an oxidative stress condition triggering sarcopenia development, finding an increased ROS production mediated by NOX2 and NOX4 (Wu et al., 2022). Notably, Wang et al. observed the effectiveness of 8 weeks of aerobic exercise in reducing NOX4 expression in skeletal muscle (Wang et al., 2022); while Qi and colleagues showed that NOX4 downregulation induced by training prevents insulin resistance and ROS production by activating the Akt signaling pathway (Qi et al., 2020). On the other hand, NOX4 has been reported by Youm et al. as a key factor in several cellular processes, including proliferation, differentiation, survival, and fusion of myoblasts, significantly contributing to muscle regeneration (Youm et al., 2019). Furthermore, NOX4 is known to promote the oxidation of glucose and fatty acids (Specht et al., 2021), as well as preserving exercise capacity and counteracting insulin resistance (Xirouchaki et al., 2021). Therefore, although this evidence collectively demonstrates how ROS produced by NOX4 play a crucial role in muscle responses to exercise and aging, its involvement requires further clarification.

Importantly, in 2011, Hori and colleagues demonstrated that silencing SIRT1 through small RNA interference in C2C12 cells led to an increase in NOX4 expression, suggesting the existence of an SIRT1-NOX4 axis (Hori et al., 2011), later confirmed by other authors (Dasgupta et al., 2020; Juan et al., 2023; Cariati et al., 2025). In agreement, we found significantly higher expression of SIRT1, a deacetylase involved in the increase of myonuclei and muscle hypertrophy (Radak et al., 2020), in the muscle tissue of the active group. This increase was associated with higher levels of PGC-1α, a known regulator of mitochondrial biogenesis, whose expression is positively modulated by SIRT1 (Chen et al., 2022). The increase of PGC-1α in active individuals could be attributed to regular exercise, consistent with findings by Dehghani et al., who reported an increase in PGC-1α mRNA in skeletal muscle of mice subjected to treadmill exercise (Dehghani et al., 2018). However, the role of PGC-1α requires the presence of ERRα, as evidenced by the involvement of the PGC-1α/ERRα transcriptional axis in improving exercise capacity (Cartoni et al., 2005). In line with this observation, significantly higher levels of ERRα were measured in the muscle tissue of the active group, suggesting that its expression might be deregulated in sedentary individuals. Finally, an upregulation of FNDC5 was observed in the muscle tissue of active women, indicating a positive exercise regulation of this factor and confirming the involvement of irisin as a mediator of the beneficial effects of physical activity (Boström et al., 2012).

### 4.3 Activation of ERRs through SLU-PP-332 treatment in myoblasts and myotubes from inactive subjects

The ability to regulate the expression of factors involved in physiological adaptations to exercise could provide a valuable opportunity to counteract both functional decline associated with aging and other pathological conditions characterized by muscle depletion. In this context, the ERRs agonist SLU-PP-332 has recently been reported as an exercise mimic capable of activating an acute aerobic exercise program, counteracting metabolic diseases, heart failure, and more generally, mitochondrial dysfunction associated with aging (Wang et al., 2023; Billon et al., 2024; Xu et al., 2024). Based on this evidence, we set up primary myoblast cultures isolated from muscle tissue of inactive subjects to examine the effects of SLU-PP-332 treatment on the expression patterns of NOX4, SIRT1, PGC-1α, ERRα, and FNDC5.

Interestingly, SLU-PP-332 treatment significantly influenced cellular metabolism by reducing LDH release, ROS production, and SA-β-gal, while increasing GSH levels. These results suggest an antioxidant action of SLU-PP-332, capable of reducing cytotoxicity and cellular senescence associated with aging. This effect might be attributed to the downregulation of NOX4 observed in myoblasts from the inactive group, further supporting its involvement in ROS production that damages skeletal muscle. Nevertheless, the conflicting evidence regarding NOX4’s role in muscle physiology calls for further investigation to clarify the importance of finely regulating the expression of this still poorly characterized factor.

An upregulation of SIRT1, PGC-1α, and ERRα was also observed in treated myoblasts, similar to that observed in untreated cells in the active group, confirming the ability of SLU-PP-332 to activate ERRs and promote cellular responses associated with exercise. These included increased expression of key factors linked to fiber hypertrophy, myonuclear growth, mitochondrial biogenesis, and cellular respiration. These findings highlight the importance of testing the efficacy of various compounds, both natural and synthetic, in promoting exercise-induced cellular responses to enhance muscle function and at least partially counteract the muscle depletion that occurs with aging.

Interestingly, treatment of myoblasts with SLU-PP-332 promoted an increase in FNDC5 expression, potentially involved in the greater fiber diameter observed in active women (Guo et al., 2023). Indeed, the reduced muscle expression of FNDC5, which is dependent on PGC-1α, has been associated with various pathological conditions, such as heart failure and obesity, suggesting the potential to counteract disorders of different origins through ERRs activation (Mozaffaritabar et al., 2024). In addition, myoblasts treated with SLU-PP-332 exhibited an upregulation of Akt and Bcl-2, indicating the inhibition of apoptotic pathways in response to ERRs activation. Finally, targeting ERRs throughout the differentiation process positively influenced myotube formation and MyHC expression, suggesting a crucial role for ERRs in muscle atrophy. This evidence confirms that activation of ERRs by treatment with SLU-PP-332 could reverse the alterations that occur in myoblasts during aging and that lead to poor myotube formation, resulting in muscle atrophy.

Overall, our findings demonstrate the crucial role of ERRs in skeletal muscle metabolism. Targeting these key regulators of muscle responses to exercise could represent a promising strategy for counteracting age-related and sedentary-induced muscle decline. Genetic knockout studies are needed to investigate the role of ERRα, ERRβ, and ERRγ in muscle responses to exercise to determine the specific contribution of ERRs to skeletal muscle health and pathology. In this context, an important first step has been taken by Fan and colleagues, who conducted single and combined muscle-specific knockout experiments in mouse models, demonstrating that PGC-1α-induced mitochondrial biogenesis is completely abolished in primary myotubes with ERRα deletion, but not ERRγ (Fan et al., 2025). Furthermore, since the expression of ERRβ in skeletal muscle is significantly lower than that of ERRα and ERRγ, a very low, if any, relevance of this receptor in muscle metabolism has been suggested (Fan et al., 2013; Billon et al., 2023). These findings suggest the importance of ERRα in muscle responses to exercise and highlight the need to further investigate the role of ERRs in skeletal muscle in health and disease. Notably, our findings support a role for ERRs in the differentiation process and in the formation of multinucleated myotubes, laying the groundwork for further studies investigating the role of these receptors in age-related muscle atrophy.

## 5 Limits of study

A limitation of our study is the small sample size, consisting of 20 elderly women undergoing hip arthroplasty surgery for coxarthrosis. The difficulty in recruiting subjects with similar characteristics in terms of age, health status, and type of surgery affected the sample size, but allowed for a more homogeneous group for greater consistency of results. In addition, the division of participants into active and inactive is based on self-reported information regarding physical activity levels. Although this methodology is common, there is a possibility of bias resulting from the subjectivity of the responses, which may not accurately reflect participants’ actual physical activity levels.

## 6 Conclusion

Our results highlight the crucial role of ERRs activation in modulating muscle responses to aging and inactivity. The SLU-PP-332 treatment in myoblasts isolated from muscle tissue of inactive subjects induced a significant improvement in cellular metabolism, reducing oxidative stress and cytotoxicity, while promoting the expression of key factors involved in muscle function, mitochondrial biogenesis, and the regulation of cellular senescence. Specifically, the downregulation of NOX4 suggests its potential involvement in the production of ROS damaging to skeletal muscle, while the up-regulation of SIRT1, PGC-1α, and ERRα confirms the effectiveness of the treatment in restoring the molecular pathways activated by exercise. Furthermore, the increase in FNDC5, together with the activation of Akt and Bcl-2 and the subsequent inhibition of apoptotic pathways, suggests a potential protective effect on muscle mass, contributing to preserving its integrity and functionality. Furthermore, the increased formation of multinucleated myotubes highlighted by the expression of MyHC through immunofluorescence analysis confirms the crucial role of ERRs in maintaining muscle mass and function during aging.

Overall, these data indicate that targeting ERRs could represent a promising approach to preserve muscle function in the elderly and in individuals with pathological conditions characterized by muscle atrophy. However, further studies will be needed to clarify the molecular mechanisms involved and assess the translational potential of this strategy. Undoubtedly, exploring the impact of ERRs activation on muscle metabolism and overall energy balance could pave the way for new targeted therapeutic strategies, offering more effective solutions to counteract sarcopenia and muscle decline.

## Data Availability

The original contributions presented in the study are included in the article/Supplementary Material, further inquiries can be directed to the corresponding author.
