# Supplementary material for: Targeting ERRs to counteract age-related muscle atrophy associated with physical inactivity: a pilot study
Source: Front Physiol. 2025 Jul 7;16:1616693. doi: 10.3389/fphys.2025.1616693 (PMC12277287; doi:10.3389/fphys.2025.1616693)
Supplement: Supplementary file 1 [file Supplementaryfile1.docx]

Supplementary Material


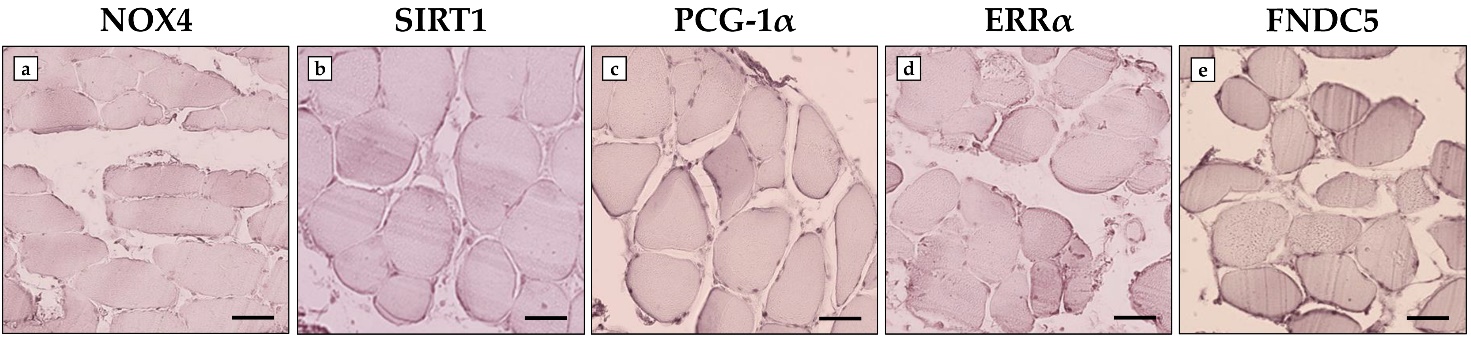


**Supplementary Figure 1. Immunohistochemistry analysis for NADPH oxidase 4 (NOX4), sirtuin 1 (SIRT1), peroxisome proliferator-activated receptor gamma coactivator 1-alpha (PGC-1α),** **estrogen-related receptor alpha (ERRα), and fibronectin type III domain-containing protein 5 (FNDC5) expression in muscle tissue.** (a) NOX4-negative control. (b) SIRT1-negative control. (c) PGC-1α-negative control. (d) ERRα-negative control. (e) FNDC5-negative control. For 40× images, scale bar represents 50 μm.


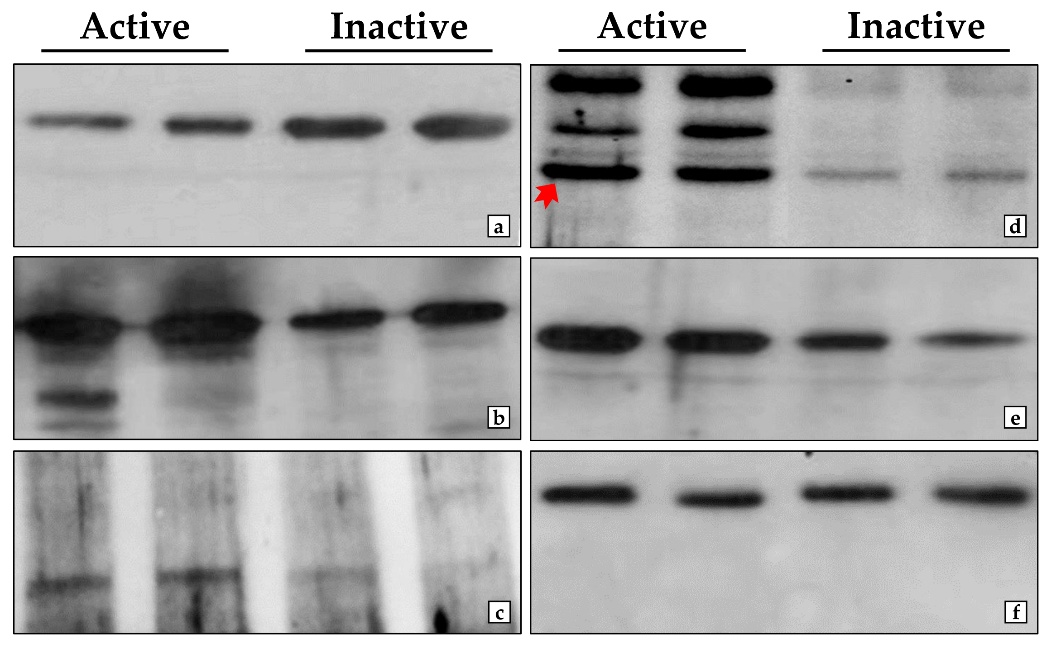


**Supplementary Figure 2. Original western blotting images for muscle tissue.** (a) NADPH Oxidase 4 (NOX4) expression (molecular weight 67 kDa). (b) Sirtuin 1 (SIRT1) expression (molecular weight 110 kDa). (c) Peroxisome proliferator-activated receptor gamma coactivator 1-alpha (PGC-1α) expression (molecular weight 91 kDa). (d) Estrogen-related receptor alpha (ERRα) expression (arrow, molecular weight 46 kDa). (e) Fibronectin type III domain-containing protein 5 (FNDC5) expression (molecular weight ~25 kDa). (f) GAPDH expression (molecular weight 36 kDa).


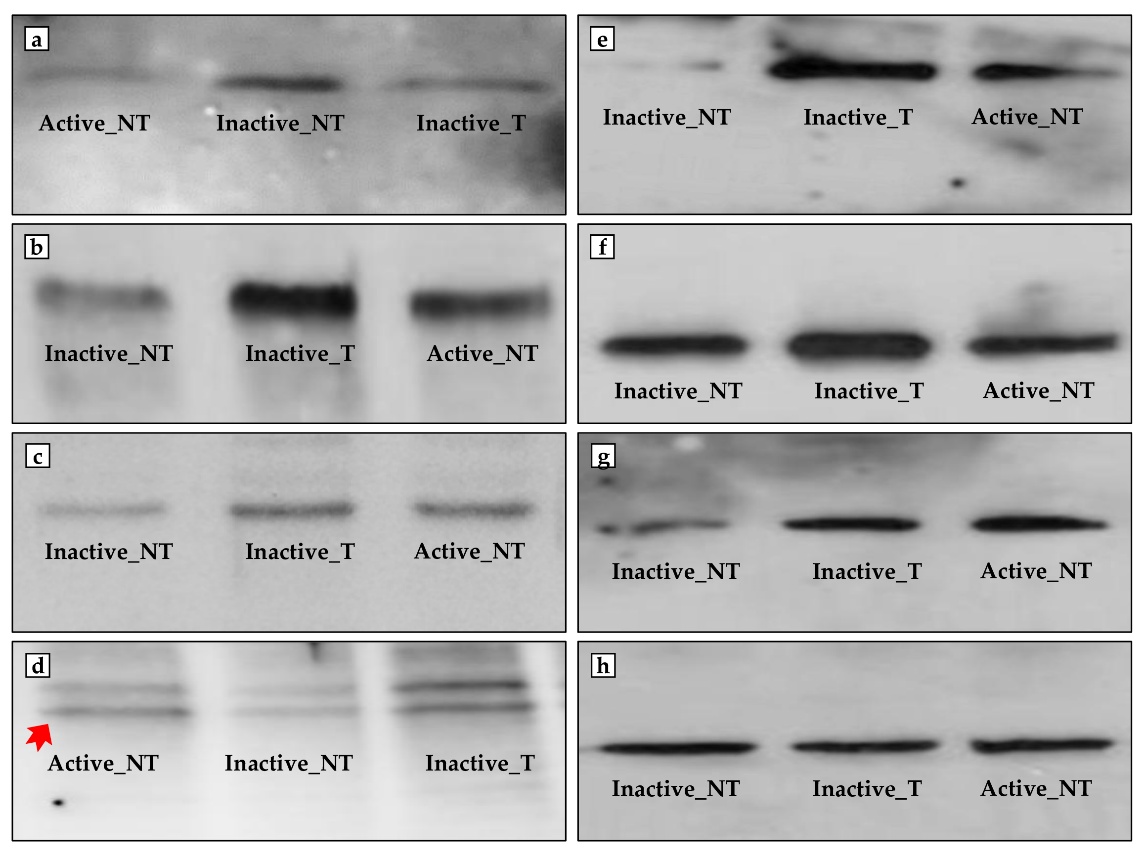


**Supplementary Figure 3. Original western blotting images for untreated myoblasts from the active group (Active_NT), untreated myoblasts from the inactive group (Inactive_NT), and myoblasts treated with SLU-PP-332 from the inactive group (Inactive_T).** (a) NADPH Oxidase 4 (NOX4) expression (molecular weight 67 kDa). (b) Sirtuin 1 (SIRT1) expression (molecular weight 110 kDa). (c) Peroxisome proliferator-activated receptor gamma coactivator 1-alpha (PGC-1α) expression (molecular weight 91 kDa). (d) Estrogen-related receptor alpha (ERRα) expression (arrow, molecular weight 46 kDa). (e) Fibronectin type III domain-containing protein 5 (FNDC5) expression (molecular weight ~25 kDa). (f) GAPDH expression (molecular weight 36 kDa).
